# Genome-based transmission modelling separates imported tuberculosis from recent transmission within an immigrant population

**DOI:** 10.1099/mgen.0.000219

**Published:** 2018-09-14

**Authors:** Diepreye Ayabina, Janne O. Ronning, Kristian Alfsnes, Nadia Debech, Ola B. Brynildsrud, Trude Arnesen, Gunnstein Norheim, Anne-Torunn Mengshoel, Rikard Rykkvin, Ulf R. Dahle, Caroline Colijn, Vegard Eldholm

**Affiliations:** ^1^​ Department of Mathematics, Imperial College London, South Kensington Campus, London SW7 2AZ, UK; ^2^​ Infection Control and Environmental Health, Norwegian Institute of Public Health, Lovisengerggata 8, 0456 Oslo, Norway

**Keywords:** tuberculosis, transmission modelling, genome sequencing, immigration

## Abstract

In many countries the incidence of tuberculosis (TB) is low and is largely shaped by immigrant populations from high-burden countries. This is the case in Norway, where more than 80 % of TB cases are found among immigrants from high-incidence countries. A variable latent period, low rates of evolution and structured social networks make separating import from within-border transmission a major conundrum to TB control efforts in many low-incidence countries. Clinical *Mycobacterium tuberculosis* isolates belonging to an unusually large genotype cluster associated with people born in the Horn of Africa have been identified in Norway over the last two decades. We modelled transmission based on whole-genome sequence data to estimate infection times for individual patients. By contrasting these estimates with time of arrival in Norway, we estimate on a case-by-case basis whether patients were likely to have been infected before or after arrival. Independent import was responsible for the majority of cases, but we estimate that about one-quarter of the patients had contracted TB in Norway. This study illuminates the transmission dynamics within an immigrant community. Our approach is broadly applicable to many settings where TB control programmes can benefit from understanding when and where patients acquired TB.

## Data Summary

Genomic short read sequences used in the current study are available under ENA study accession PRJEB23495. Additional information on samples and epidemiology is available as supplementary material. Data on immigration to Norway were extracted from Statistics Norway (https://www.ssb.no/statbank/table/07822/).

**Impact Statement**

In many countries, tuberculosis (TB) is uncommon in the native-born population. In such settings, immigrants from regions with a much higher TB burden may constitute a majority of TB patients. TB infections can lie dormant and undiagnosed for years and even decades, before occasionally causing disease. Together with the very low evolutionary rate of the *Mycobacterium tuberculosis* genome, which lowers the resolution of genome sequence-based comparisons of clinical TB isolates, this significantly complicates efforts to map transmission. Additional difficulties arise when TB patients are immigrants from countries with a high incidence of TB, as the patient could have been infected either in the origin country, en route or after arrival in the receiving country. Identifying active transmission chains is a central activity of efforts to halt TB transmission. In this study of a widespread TB strain among Norwegian immigrants from the Horn of Africa, we combined genome sequences of clinical TB isolates with epidemiological data as input for epidemiological modelling. This allowed us to estimate the approximate time of infection for individual patients, which was then compared to their time of arrival in Norway. This novel approach proved helpful for separating imported TB incidents from infections acquired in Norway. The method is broadly applicable, but requires significant resources.

## Introduction

The ‘End TB strategy’ of the World Health Organization aims to reduce the global incidence of tuberculosis (TB) to 100 cases per million by 2035. Reaching this goal entails reducing the global TB incidence to the rates currently seen in countries with the lowest incidence, whereas low-incidence countries must aim for further reductions in incidence levels.

As the TB epidemic is fading out of the local population, its incidence in Norway now largely reflects the level of immigration from countries with a high incidence of TB [1–3]. With effective case finding and case management, TB transmission from immigrant populations to the Norwegian-born population has been found to be very limited [4], but it does occasionally occur [5]. In low-incidence countries, preventing transmission within immigrant groups originating from high-incidence countries is of utmost importance if the overall incidence of TB is to be reduced further.

Detecting transmission within immigrant populations remains a complicated task as it requires the ability to distinguish between import and recent transmission in the receiving country. Even when genome sequences are available for analysis, the reconstruction of detailed transmission histories is far from straightforward. As a result of a low evolutionary rate and highly variable latency periods, this is especially true for TB [6, 7]. This is further complicated by social networks that are partially shaped by shared cultural and ethnic backgrounds [8]. *Mycobacterium tuberculosis* genotypes that are circulating in the region of origin of individual immigrant groups can appear to be outbreaks in a receiving country where strains are more diverse and there is less overall transmission. It can be difficult to determine whether these are true outbreaks, reflecting recent transmission, or repeated importation.

Long-distance migration is in itself stressful and could be a driver of TB transmission, as it could trigger reactivation of latent disease and often entails long-term detention in crowded centres. A study of TB patients having recently arrived in Europe from the Horn of Africa concluded that the patients had probably contracted the disease in detention centres en route [9].

Due to low discriminatory power, genotyping methods based on *IS6110* RFLP or mycobacterial interspered repetitive units (MIRU) tend to over-estimate recent transmission. Increased accuracy is, however, attainable by introducing epidemiological constraints, such as requiring that a source case is identified (the identification of a matching genotype in a patient diagnosed within a relevant temporal and geographical window) for a case to be defined as one of recent transmission [10].

By employing such field-validated methods, recent transmission was found to have caused 14 % of TB cases in the United States in the period 2011–2014, compared to 22 % when relying on genotyping alone [11]. These approaches are probably relatively robust, but are laborious due to the requirement of extensive contact tracing efforts, and ultimately are limited by the low accuracy of RFLP and MIRU profiling [5, 12]

Several approaches have been developed to make use of genomic data to infer infector/infectee relationships. They differ in their statistical approaches, the complexity of the underlying epidemiological models and the assumptions they make about unsampled cases, transmission bottlenecks, pathogen evolution, diversity inside hosts and the likelihood of transmission events. Key parameters that must be specified or estimated include the generation time (time from an individual becoming infected until infecting others) and patient and health system delay (time from symptom onset to diagnosis). In addition, inference requires timing information and a model of the pathogen's evolution to connect the acquisition of polymorphisms to the time that has elapsed [13–17].

Didelot *et al.* developed a method to infer transmission networks from time-labelled phylogenies assuming a susceptible–infectious–removed (SIR) epidemiological model [13]. Building on the same approach, Eldholm *et al*. implemented a latent state (‘Exposed’ category) in a similar framework to estimate the probability of pairs of patients being linked by a transmission event [7]. TransPhylo [6] is a Bayesian method for inference of transmission trees that also accounts for unsampled cases and infers transmission events and their timing given mutational events captured in a time-labelled phylogeny; it uses a branching model rather than an SIR or susceptible-exposed-infectious-removed (SEIR) framework.

Here, we apply a novel approach to investigate whether immigrants diagnosed with TB represent imported cases or local transmission. We focused on a large genotypic *M. tuberculosis* cluster (Norwegian–African large Lineage 3 cluster; NAL3C), strongly associated with immigrants from the Horn of Africa, that has been identified in Norway consistently for almost 20 years [18]. Building on a temporal phylogeny reconstructed from genome-wide SNPs, we infer the posterior distribution of infection times using TransPhylo. We then compare these with the time of arrival of individual patients in Norway, to ascertain probabilistically whether they became infected before or after their arrival in Norway. With this hybrid method, which relies on a combination of genome sequence data, epidemiological data and transmission modelling, we estimate that about 25 % of the patients probably contracted TB after arrival in Norway.

## Methods

### Sample collection and epidemiological data

The NRLM maintains a national culture collection consisting of all culture-positive TB cases in Norway and is responsible for susceptibility testing and genotyping. From 1997 to 2010 *IS6110*-RFLP was the routine method for molecular epidemiological surveillance. In this period, a large *IS6110*-RFLP cluster associated with patients from the Horn of Africa was identified [18]. Following the replacement of *IS6110-*RFLP typing with 24-loci MIRU genotyping in 2011, these isolates were re-typed and the majority of isolates belonged to the MIRU type 1064-32 based on the MTBC 15-9 nomenclature [19]. All subsequent *M. tuberculosis* isolates have been MIRU typed. In order to study the transmission dynamics of this cluster, we included all isolates sampled between 1997 and 2015 that differed at zero to two loci relative to the 1064-32 genotype. In total, 133 isolates matched the inclusion criteria, of which 130 could be retrieved and were submitted to whole-genome sequencing on the Illumina platform. Initial analyses revealed one of these to be a clear outlier only distantly related to the other 129 isolates; it was thus excluded. The 129 isolates represented 127 patients (three isolates were from the same patient). The cluster, as defined by *IS6110*-RFLP genotyping, was recently termed ‘Cluster X’ [18]. However, we coined the more informative term NAL3C for the current study.

Minimal epidemiological data were extracted from the Norwegian Surveillance System for Communicable Diseases (MSIS), which stores clinical and epidemiological data on all TB cases notified by clinicians.

### Variant calling and phylogenetic analyses

DNA was extracted from *M. tuberculosis* grown on Lowenstein–Jensen slants as described previously [5]. Paired-end sequences were generated on the Illumina MiSeq and NextSeq platforms (250 and 150 bp read length, respectively). High-quality SNPs were identified following the same procedures as described previously [5]. Briefly, SNPs in or within 50 bp of regions annotated as PE (Pro-Glu) or PPE (Pro–Pro–Glu) genes, mobile elements or repeat regions were excluded from all analyses. Heterozygous SNPs that were found at a frequency of 10–90 % of reads in at least one isolate were also excluded. For inclusion of SNPs, a minimum depth of 10 reads in one genome and at least four reads in all strains was required. After removal of the single outlier isolate, this resulted in 1418 variable sites that were used for evolutionary analyses as outlined below. Median sequencing depth of the 129 genomes ranged from 20× to 161×. All sequence reads are available under ENA study accession PRJEB23495. Individual run accessions, sampling years and MIRU data for all NAL3C isolates are listed in Supplementary Dataset 1.

Sampling dates were used to calibrate a temporal phylogeny generated with beast 1.8.4 [20], and tip-randomization performed to verify the presence of a robust temporal signal (Fig. S1, available in the online version of this article). Marginal likelihood estimates were performed to identify the optimal substitution, clock and demographic models for Bayesian evolutionary analyses. A GTR model with relaxed clock combined with a Skyride demographic model was favoured (Table S1).

### Transmission reconstruction

**Fig. 1.**
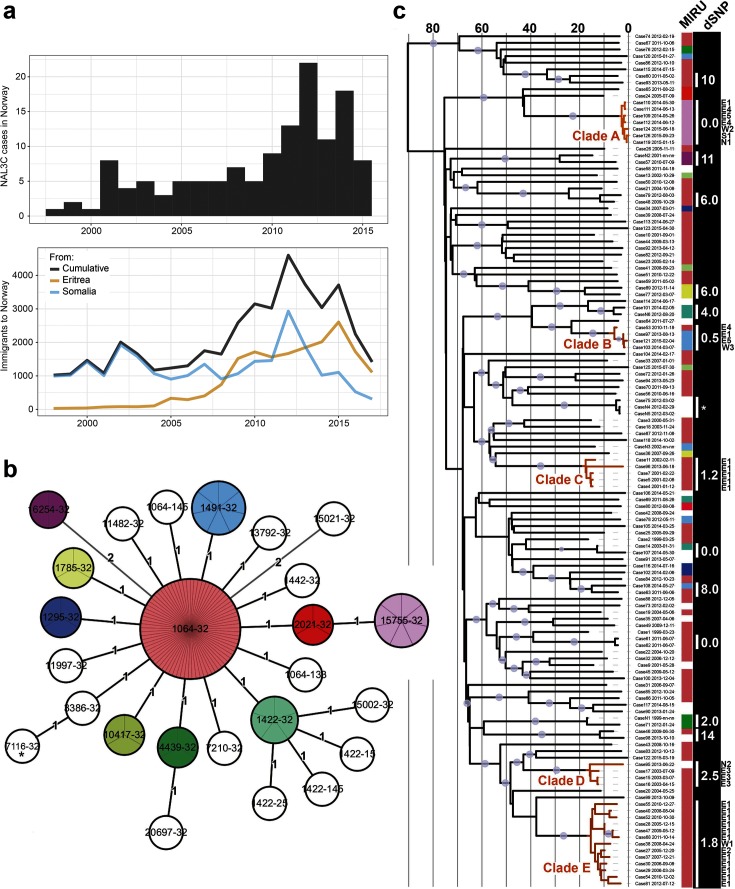
Clinical *M. tuberculosis* isolates and phylogenetic reconstruction. (a) Top panel: histogram of sampling times for NAL3C isolates. Bottom panel: immigration from Eritrea and Somalia to Norway over the same time span. (b) NAL3C minimum-spanning tree based on 24-loci MIRU genotypes. MIRU profiles identified in more than one isolate were assigned individual colours. (c) Temporal phylogeny reconstructed from genome-wide SNPs. The colour strip next to the phylogeny indicates MIRU genotype, whereas the dSNP column denotes mean pairwise SNP distances within each cluster. Clades amenable for TransPhylo transmission reconstruction are highlighted in orange. The time axis on the phylogeny corresponds to years before 2015. An asterisk denotes three samples isolated from the same patient. Grey dots on branches indicate posterior probability of >0.8. The county of residence of patients belonging to clades A–E is annotated in the right margin (see Methods for details).

The maximum credibility tree of the 129 isolates was characterized by long branch lengths with a few clades that have relatively short branch lengths (Fig. 1). As the branch lengths of a timed phylogenetic tree represent the duration of evolution [21], we assumed that these clades represent densely sampled clusters of cases whereas the long branches represent cases with unsampled infectors. We thus reasoned that putative transmission clusters in Norway would be represented by sub-clades of closely related isolates within the larger NAL3C cluster. As the vast majority of immigrants from the Horn of Africa came to Norway after 1995, following withdrawal of the United Nations from Somalia, we only considered clades with an inferred most recent ancestor younger than 20 years (corresponding to 1995). As a tree must include at least four cases for meaningful modelling in TransPhylo, this inclusion criterion was also applied.

A total of five clades (clades A, B, C, D and E shown in Fig. 1) met the criteria for detailed transmission modelling. There are a total of 33 cases in the selected clades, with times of arrival in Norway available for 22 of them. In addition, manual transmission inference was performed on pairs and triplets of closely related isolates. TransPhylo requires priors for the generation time and time to sampling; these were estimated from data using all cases in one of the clades (clade A); the fitted posterior values (Figs. S2 and S3) were used to specify the generation time and sampling time densities in the onward analysis. We explored a range of prior distributions (Table S2 and Fig. S4). Within this range the key results regarding recent transmission are not dependent on the choice of prior. To reflect the fact that smear-negative patients are likely to be less infectious than smear-positive patients, we modelled transmissions from smear-negative patients as 75 % as likely as transmissions from smear-positive patients (all else being equal); results on recent transmission are not altered when this penalty is removed. The rationale for this choice of penalty is that while it is thought that smear-negative patients are less infectious than others, there is evidence that a minority of transmissions are due to smear-negative cases in some populations in low-incidence countries [22].

TransPhylo is a ‘two-step’ method, relying on first reconstructing a timed phylogenetic tree and then performing Bayesian inference of the transmission events. We accounted for phylogenetic uncertainy by repeating the inference on different posterior timed phylogenetic trees (see Supplementary methods and results, Figs S5 and S6). TransPhylo requires assumptions about the parameters *r* and *p* (negative binomial offspring distribution parameters), the in-host effective population size *N*_e_*g* and the overall sampling probability *pi*. We fixed *N*_e_*g* and assumed a mean offspring of 1 and a sampling fraction of 0.95 (see Supplementary methods and results). Transmission trees based on the median values of posterior distributions are presented in Fig. S7.

### Geographical information in Norway

Norway is divided into 19 counties. Here we do not use county names in order to minimize the use of possibly sensitive data, but rather refer to these counties as E (East), S (South), W (West) and N (North) followed by a number unique to each county.

Please see the Supplementary Methods for a full description of all methods employed.

## Results

### Quantifying transmission in Norway

Based on 24-loci MIRU typing, 129 clinical *M. tuberculosis* NAL3C isolates from 127 patients, collected between 1997 and 2015 (Fig. 1a, b) were sequenced at the Norwegian National Reference Laboratory for Mycobacteria (NRLM). A total of 1418 variable sites were identified, resulting in a mean pairwise SNP distance of 43.22 separating the NAL3C isolates. A temporal phylogeny was estimated in beast 1.8.4 [20] utilizing sampling dates for temporal calibration (Fig. 1c).

Comparing the MIRU-based minimum-spanning network and the whole-genome phylogeny of our samples (Fig. 1), it was clear that the true genomic diversity of the NAL3C cluster was far from completely captured by MIRU typing. Strikingly, we also find that the micro-evolution of MIRU loci within the cluster evolved in a way that was not informative for molecular epidemiological purposes. Indeed, there is evidence that lineage strongly affects how well MIRU similarity predicts whole-genome sequence similarity [23]; this clearly affects the interpretation of MIRU data.

The high genetic diversity within the NAL3C cluster, combined with an overall phylogenetic structure characterized by multiple long terminal branches interspersed by a handful of tight clusters, suggested that the clinical TB cases in Norway represented samples drawn from a larger population of mainly unsampled cases circulating in the Horn of Africa. This notion was further supported by the degree to which NAL3C incidence in Norway mirrored the load of immigration from Eritrea and Somalia (constituting the two largest immigrant groups from the Horn of Africa in Norway) (Fig. 1a). Applying the inclusion criteria described in the Methods section resulted in a total of five clades (clades A, B, C, D and E shown in Fig. 1) meeting the criteria for detailed transmission modelling. Most of the cases in these clades come from countries in the Horn of Africa, two from Sudan and one case each from Norway, two West African, one West Asian and one East Asian country.

**Fig. 2.**
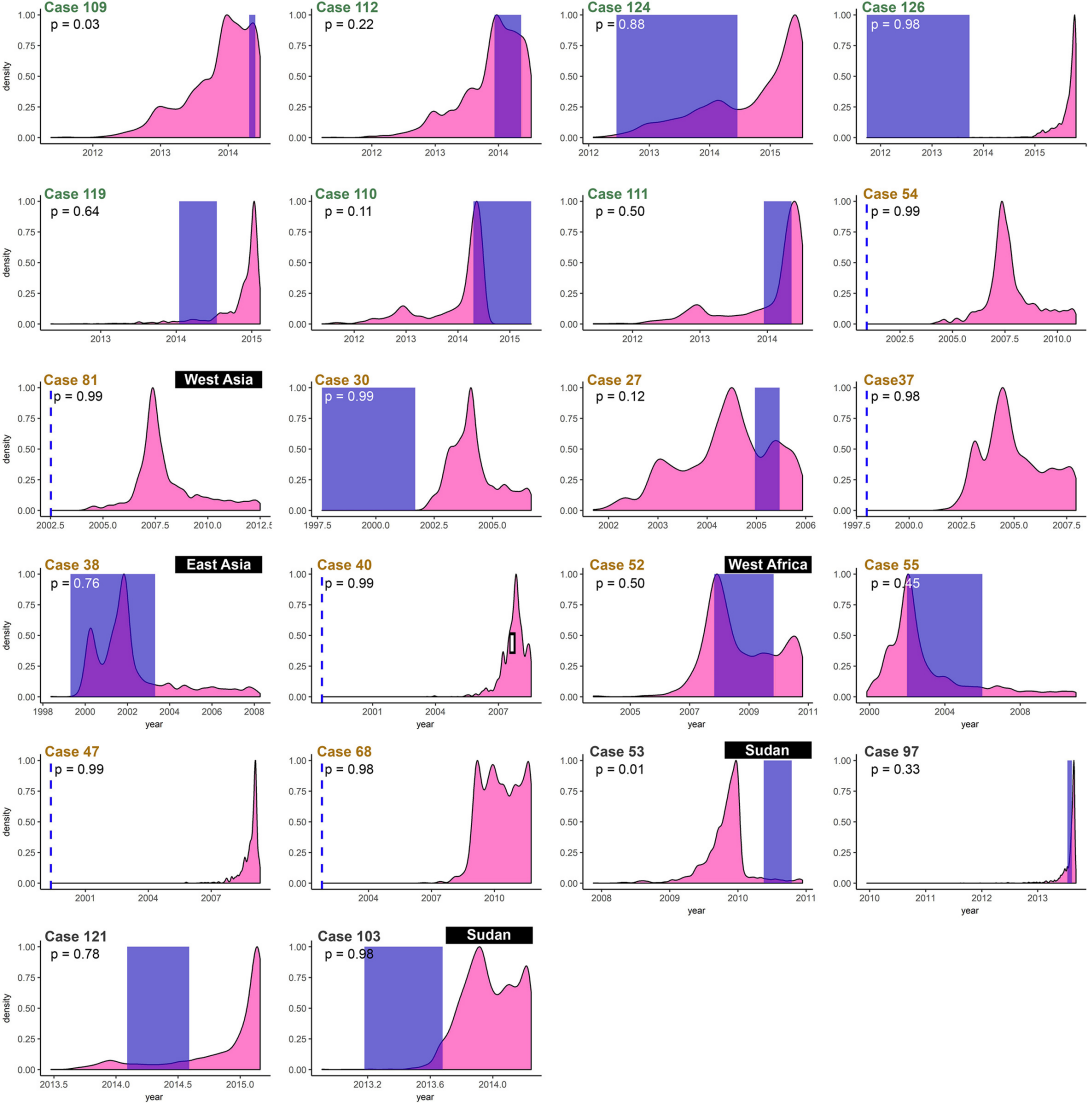
Arrival times (in blue) plotted with estimated infection times for all cases of interest with available data. The case numbers are coloured by clade assignment (clade A in green, clade E in orange and clade B in grey). The blue shaded area covers the time from earliest and latest possible arrival times, whereas a dotted single line indicates the latest possible arrival time. The listed probabilities indicate the probability of infection after arrival in Norway, averaged over 10 different TransPhylo inference procedures. The country of origin of patients not originating from the Horn of Africa is annotated in black boxes.

Fig. 2 shows the arrival times of all cases from these clades for whom arrival times were retrievable, plotted on top of the posterior infection time distribution for each individual (see Methods and Supplementary methods). It is clear that some of these patients (cases 30, 37, 40, 47, 54, 68 and 126) arrived in Norway before the estimated infection time (*T*_inf_). For all other cases, the estimated range for the time of infection has at least some overlap with the time of arrival. Using the posterior densities of infection times alongside the arrival times of the cases, we obtain probabilities of infection after the time of arrival in Norway [*P*(*t*_arrv_); see Table S3].

**Fig. 3.**
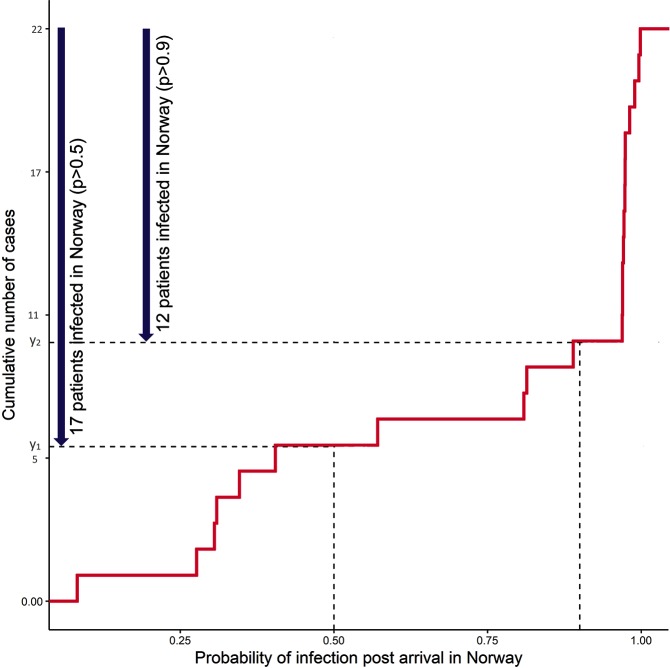
Probability of infection after arrival in Norway for 22 cases included in TransPhylo analyses. The lines indicate the number of cases with probabilities equal to 0.5 and 0.9 in the cumulative distribution plot.

The cumulative frequency plot of these probabilities (Fig. 3) shows that there are 17 cases with *P*(*t*_inf_ after *t*_arrv_)>0.5 (Table 1) and 12 cases with *P*(*t*_inf_ after *t*_arrv_)>0.9.

**Table 1.** Summary of transmission inference TP, TransPhylo inference; na, not applicable.

Inference type	Yes	Undetermined	No	Clades
TP (with arrival info)	17	0	5	A, B, C
TP (without arrival info)	7	4	0	C, D, E
Pairs and triplets	3	6	6	–
Other cases	0	0	79	–
Total (%)	27 (21)	10 (8)	90 (71)	na

In clades A, B and E, cases 28 and 29 lacked arrival time information. For case 29 (Somali) we concluded that the patient probably contracted TB in Norway, as the inferred infector was also infected in Norway. The genome of the isolate from case 28, an immigrant from West Africa, was identical to the one from case 29, indicating that case 28 was also infected in Norway.

Next, we looked into clades C and D for which arrival information was lacking for all isolates. For clade C, TransPhylo inferred that the same unsampled case had infected both a Norwegian, an Ethiopian and two Somali patients, as well as a final Somali patient via another unsampled intermediate (see Fig. S7), although we note that with long and variable infectious periods, as is the case for TB, considerable uncertainty remains in the details of reconstructed transmission events. However, our results suggest that all five of these patients contracted TB in Norway. In clade D, all patients were Somali. This, combined with a lack of arrival information for these patients, makes it impossible to distinguish between transmission before or after arrival.

In order to obtain a more complete picture of transmission in Norway, beyond clades that were amenable to transmission inference using TransPhylo, we manually investigated the temporal phylogeny for pairs and triplets of closely related isolates for evidence of transmission in Norway. Following the inclusion criteria applied for TransPhylo inference, we only included pairs and triplets with an estimated most recent common ancestor after 1995. Based on a combination of arrival times, disease manifestation and country of origin, we were able to identify another three instances of very probable transmission in Norway. For five cases we concluded that transmission in Norway was highly unlikely, whereas no conclusion could be drawn for six of the cases (see Table 1 and Tables S4 and S5 for a summary of the evidence).

In total, we concluded that 27 out of 129 NAL3C cases were probably infected in Norway. For 10 cases we were unable to make a conclusion, whereas the remaining 90 probably represented instances of imported TB (Table 1).

Retrospectively, we retrieved available contact tracing information for cases belonging to clades A, B and E. These data were not used to inform transmission reconstruction, but were nonetheless interesting to investigate as an independent check on inferred transmission events. The data were incomplete, but useful information was available for six of the cases. For five of these patients the extended data supported our inference: three cases estimated as probably infected in Norway had known TB contacts in Norway (cases 40, 47 and 81); the estimated transmission time concurred with an earlier episode of TB (case 55); and in one case a negative TB screen upon arrival in Norway was consistent with our inference of infection after arrival (case 103). However, in one case (case 119) we estimated infection in Norway but the patient reported to have had an episode of symptomatic, untreated TB before arrival; the recent isolate could reflect a re-infection, or our estimation could be incorrect.

To shed additional light on where transmission occurs, we investigated our inferences in the context of where individual patients lived in Norway at the time of diagnosis. The seven patients in clade A were all of Eritrean origin (Table S3), but were scattered between six different counties covering large parts of Norway at the time of diagnosis. This suggests that those patients inferred to have been infected in Norway were probably infected en route or soon after arrival to a common destination in Norway such as a reception centre. Unfortunately no further information is available to clarify this. The four patients in clade B were from three nations (Table S3) and lived in four different counties, again suggesting a reception centre as a likely place of infection. Nine out of 11 patients in clade E lived in the same county (E1) and one in a neighbouring county (E2), suggesting transmission in the community. The five patients in clade C were from three different nations, and all lived in county E1 at the time of diagnosis, supporting our inference of transmission in Norway (Table S5). Finally, in clade D, where no conclusions regarding transmission in Norway could be made (Table S5), three of four patients lived in county E3, which does suggest that transmission in Norway could be responsible for some of the cases.

### Comparing the hybrid method to a distance-based approach

**Fig. 4.**
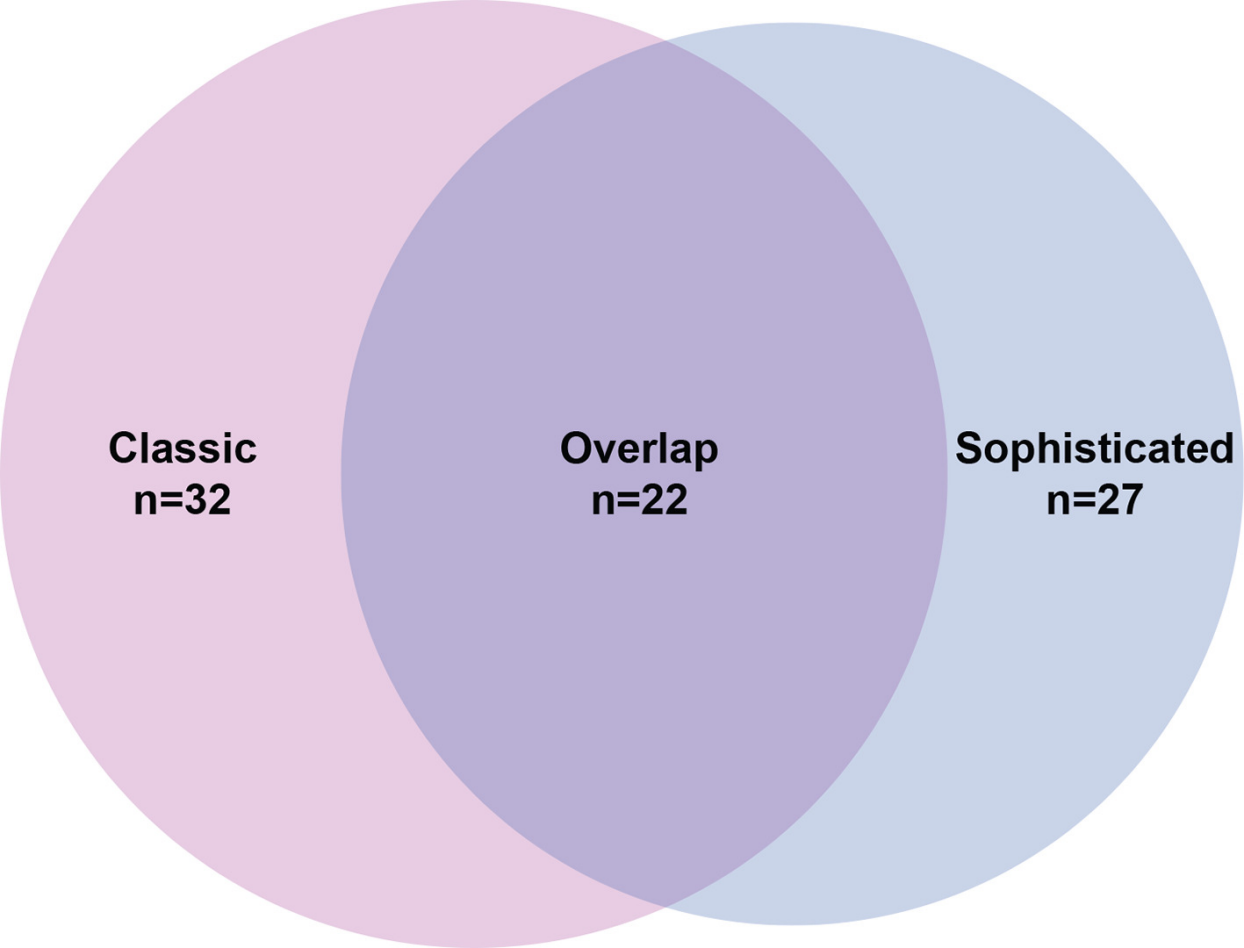
Venn diagram of cases identified as being the result of recent transmission in Norway, applying two dfferent approaches: the sophisticated approach described in the current study (Sophisticated) and an approach based on pairwise SNP distances (Classic).

Finally, we compared the results obtained with our hybrid method, which relies on a combination of genome sequence data, epidemiological data and transmission modelling, with inferences employing a simpler genomic distance-based approach [24]. In a retrospective observational study by Walker *et al.*, it was observed that the vast majority of pairs of isolates from patients with known epidemiological links were separated by fewer than six and rarely more than 12 SNPs [24]. Thus, an SNP distance of less than six between pairs of isolates can be interpreted as a confirmation of recent transmission, whereas a distance of 6–12 SNPs remains undetermined (but note that this approach is relatively crude, and bound to occasionally lead to erroneous conclusions). In the current context, it would also be reasonable to regard the first case of any pair or cluster of genomically linked cases (separated by fewer than six SNPs) as imported, and subsequent cases as transmission in Norway. Applying these criteria, a total of 32 cases were identified as likely cases of recent transmission in Norway with the simpler method, versus 27 with the hybrid method, with 22 cases identified by both methods (Fig. 4).

In the case of pairs with a distance between them of fewer than six SNPs, it would not make sense to rule out transmission based on country of origin of patients (a person might well have transmitted TB to a secondary case in Norway, despite both being from the same country). However, for pairs with an SNP distance of 6–12 SNPs it would make sense to conclude recent transmission in Norway if the patients originated from different countries. However, among six pairs of cases differing by 6–12 SNPs, thus representing six putative cases of import and six putative cases of recent transmission, all pairs of cases originated in the same country, except one pair where the origin of one patient was unknown. In other words, geographical information did not allow any refinement of the inference based on a classical approach in this case.

Among the 11 cases specifically ruled out by the sophisticated analyses (either based on the time of arrival versus inferred time of transmission or based on the transmissibility of the putative infector), four were also ruled out with the classic approach as they were the first cases in their individual cluster. Five were identified as recent transmission and two remained undetermined.

## Discussion

The current study highlighted the limited power of MIRU typing for outbreak investigations. We observed that a number of homoplasic events led to the repeated evolution of identical MIRU types across the NAL3C (Fig. 1). Based on these analyses it is clear that MIRU typing worked rather well for crude grouping of isolates, i.e. to define inclusion criteria for whole-genome sequencing, but that micro-evolutionary events, such as the mutation of a single MIRU locus, are not necessarily informative for molecular epidemiological inference. NAL3C belongs to lineage 3, an understudied *M. tuberculosis* lineage. The degree to which the mode and rate of MIRU evolution differs between lineages [23] is a question that deserves attention.

In Norway, contact tracing is initiated around all pulmonary TB cases, but the intensity of the effort is higher when recent transmission is suspected. The bar for initiating broad contact tracing efforts will thus often be higher for TB cases belonging to high-risk groups such as immigrants from high-incidence countries. However, we found that there is substantial evidence of ongoing transmission in Norway. Our findings thus highlight the importance of active contact-tracing in high-risk groups in low-incidence countries.

There are a number of limitations to our approach. While we took steps to accommodate phylogenetic uncertainty by repeating our analysis starting with different input trees, we do not have a way to compare parameters such as within-host diversity directly to data, nor can we directly validate the inferred times between infection and sampling, or between infection and infecting others, against data. This is a fundamental limitation in any outbreak reconstruction task, as the ground truth is not known. However, our results on the timing of infection are robust to different input phylogenies and choices of priors. Similarly, we do not know the contribution of smear-negative individuals to transmission, nor, indeed, the constancy of smear status over the course of infection. We used a penalty on transmission by smear-negative cases because they are widely believed to be less infectious than smear-positive individuals; our conclusions that there is evidence of TB transmission in Norway, and for whom, are not affected by removing this penalty.

We did not have individual-level data on human immunodeficiency virus co-infection or other factors that may have promoted TB disease, so we cannot draw conclusions about host factors that may have caused rapid disease progression. We also note that by the nature of the study, we are not finding and including individuals who have ‘latent’ (asymptomatic, contained) TB; only those with active disease would produce culture-positive samples that can be sequenced, and TransPhylo only augments the tree with unsampled individuals who are in transmission chains with sampled cases. The model does not include unsampled individuals who remain latent or uninfectious, or who are infectious but do not infect other individuals so as to be ancestral to the sampled cases. Accordingly, the generation times in the model reflect generation times for individuals who progress to active disease by the time of sampling, and who have done so in time to infect others who have progressed to active disease and arisen in transmission chains in our inference. Even in this defined set of unsampled cases, there may be a systematic difference between sampled and unsampled cases, for example if unsampled cases are unsampled because they do not seek health care and if this correlates with higher transmissibility, higher bacterial burden, shorter generation times or other parameters. TransPhylo does not account for this bias, and it would be challenging to determine its nature without data on unsampled cases. Finally, TransPhylo does not model a contraction of generation times as an epidemic proceeds [25]; the branching process model is built on the approximation that clustering is relatively low (individuals are unlikely to expose each others’ contacts) and that the susceptible population size is much larger than the infected population. The latter is very reasonable in this context where TB rates are very low. While there may be social clustering in close-knit immigrant communities, the sparseness and lack of community-based clustering in the full phylogeny suggest that low clustering is a reasonable assumption. TransPhylo also carries the advantage that it handles unsampled individuals and within-host diversity.

Even without explicitly reconstructing transmission events, some conclusions about the timing of transmission can be drawn from the timed phylogenetic tree. Each tip of the tree corresponds to a different host, which implies that there must be at least one transmission event on the path between every pair of tips [6, 13]. Recent branching in the timed phylogeny therefore indicates recent infection. Our analysis goes further, by producing probability distributions for infection times for individual patients; this was achieved by combining genome sequences, clinical data and epidemiological data to inform transmission modelling. We believe this work represents a conceptually novel and useful approach to tackle an important public health issue in countries of low TB incidence.

We find that 27 patients belonging to the NAL3C cluster were most probably infected in Norway, whereas 90 patients had probably contracted TB prior to arrival in Norway. For 10 patients, our analyses were inconclusive. In addition, we were unable to retrieve samples for DNA extraction for three patients, originating from Norway, Eastern Europe and Southern Africa respectively. Based on their country of origin alone, these were all probably infected in Norway. Only considering patients for whom a conclusion could be drawn, about one-quarter were most likely to have contracted TB in Norway, a far from trivial proportion. It should be noted that some patients might also have been infected during travels to the country of origin after arrival in Norway, but we were not in a position to investigate this possible order of events.

A very recent study from the Netherlands and Denmark identified the 1064-32 MIRU-type among refugees and immigrants with a similar country profile as observed in Norway [26]. Whole genome sequencing of 40 isolates revealed a pairwise SNP distance of 80, almost twice as high as observed here. Although formal transmission reconstruction was not performed, the high diversity supports their conclusion that transmission in the Netherlands and Denmark is very limited. The lower diversity in Norway also suggests that recent transmission here has affected the observed population structure of NAL3C.

### Conclusion

We show that transmission modelling based on a combination of clinical, epidemiological and genomic data can assist public health authorities in understanding where and when patients are infected, and can aid in the design of appropriate TB control measures. A major novelty in the current work concerns contrasting time of immigration with infection times as inferred by transmission modelling. This allowed us to formally estimate the degree of recent transmission versus repeated import of an unusually large genotype cluster associated with immigrants from the Horn of Africa. Importantly, the use of genomic and epidemiological data to quantify local transmission can thus serve as a useful metric to gauge the quality of local/national TB programmes.

## Data bibliography

1. . Ayabina D, Rønning JO, Alfsnes K, Debech N, Brynildsrud OB *et al.* Genome-based transmission modeling separates imported tuberculosis from recent transmission within an immigrant population PRJEB23495 2018.
2. . Statistics Norway. 2018. https://www.ssb.no/statbank/table/07822/ [accessed July 2018].

## Supplementary Data

Supplementary File 1

Supplementary File 2
