# Supplementary material for: Genome-based transmission modelling separates imported tuberculosis from recent transmission within an immigrant population
Source: Microb Genom. 2018 Sep 14;4(10):e000219. doi: 10.1099/mgen.0.000219 (PMC6249437; doi:10.1099/mgen.0.000219)
Supplement: Supplementary File 1 [file mgen-4-219-s001.pdf]

Supplementary Methods and Results

August 23, 2018

Bayesian Evolutionary analyses and test of temporal signal

Marginal likelihood estimates in Beast 1.8.4 [3] were performed to identify the optimal substitution, clock and demographic models for Bayesian evolutionary analyses. We tested the HKY and GTR substitution models combined with either a strict or uncorrelated relaxed clock and a constant, logistic, exponential or Skyride demographic model. A GTR model with relaxed clock combined with a Skyride demographic model was favored (Table S1). Three independent Markov chain Monte Carlo (MCMC) chains consisting of 200 million steps were performed and the output combined after inspection of convergence within and between chains. These analyses resulted in an estimated substitution rate of 8.99E-8 (95 per cent HPD: 5.07E-8, 1.31E-7) substitutions per genome per year. To verify the presence of sufficient temporal signal in the data, tip-randomization was performed utilizing the 'tipdatingbeast' R package [9]. Of 20 tip-randomized runs, the 95 % HPD interval of a single run overlapped with the tree height 95% HPD interval generated in the combined non-randomized data , indicating that the strength of the temporal signal was acceptable [4, 8].

TransPhylo inference

We used the R package TransPhylo [1] to reconstruct the outbreaks. TransPhylo allows for in-host diversity; individuals may harbour more than one pathogen variant (though they may not). The within-host model has a parameter $N_e g$, such that pathogen lineages within a host coalesce at a rate $N_e g$. The underlying epidemiological model is a stochastic branching process, in which the number of secondary infections follows a negative binomial offspring distribution, and the user sets a prior for the serial interval γ (the probabilistic time between an individual becoming infected and infecting others) and for the sampling interval σ (the time between becoming infected and becoming known to the health care system and having a sample taken). This captures variability in host contact patterns and infectiveness. The duration of infectiousness is permitted to be highly variable; in practice this is done by choosing γ to have high variability. The epidemiological model requires the user to specify a gamma distribution for the generation time, and similarly, a gamma distribution for the sampling time. Estimating these parameters is challenging when the sampling density is unknown. However, for the five sub-clades identified above, we can assume a high sampling density based on the extremely limited observed diversity within each clade. We estimated the parameters of the sampling and generation time distributions from the subtree of the least diverse clade (clade A; mean pairwise SNP-distance = 0), assuming 95% sampling.

For the transmission inference in TransPhylo, we chose parameters of the negative binomial distribution that would give a reproduction number of 1. This relatively low number is expected for tuberculosis in a low burden, setting like Norway

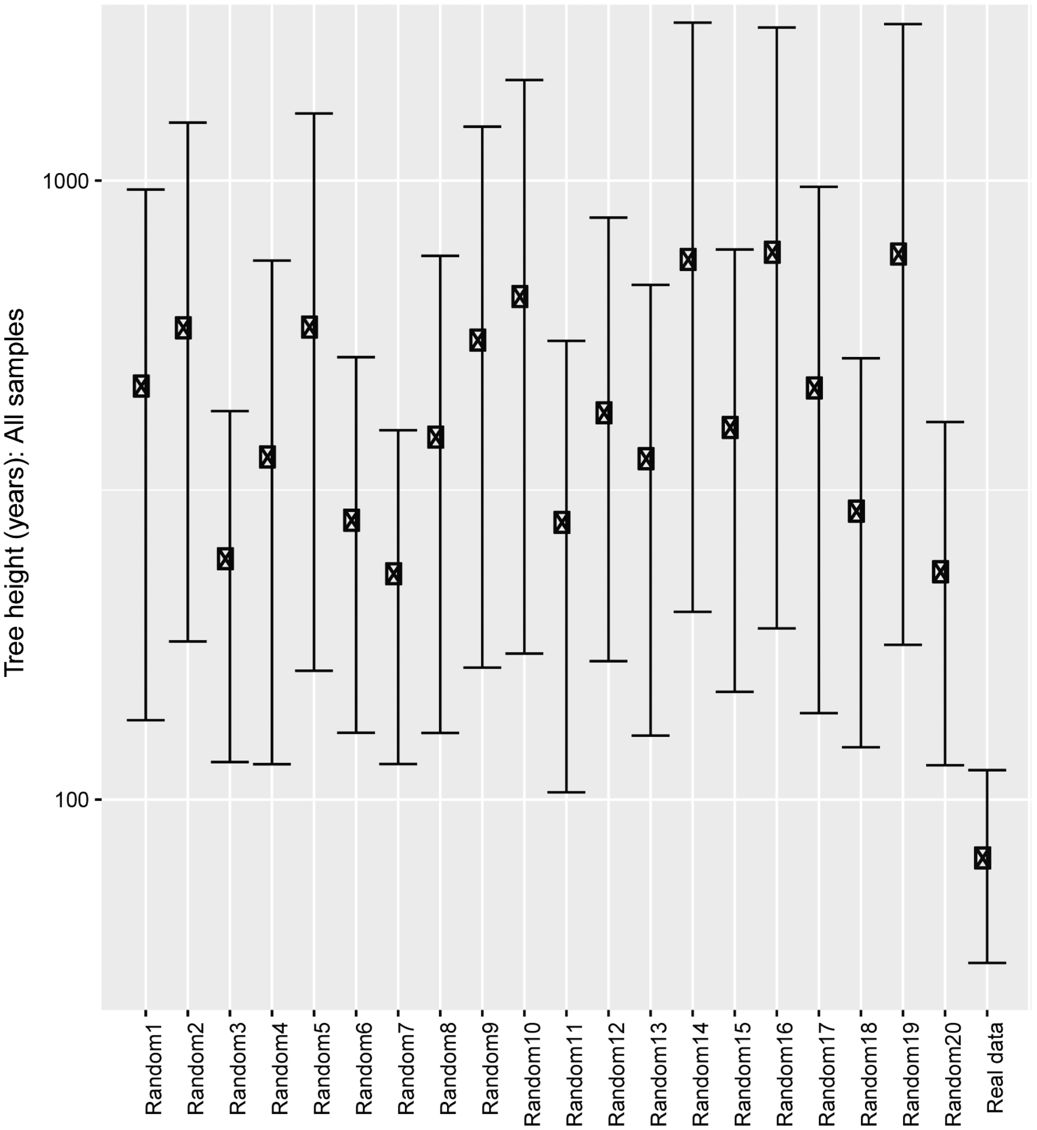

Figure S1: *Tree height estimated after randomization of tip-dates. Height estimated from the real non-randomized sampling dates can be seen to the right. In one of the 20 randomized runs, the 95 per cent HPD for tree height overlapped with the estimates from the real data.*

[5]. Also we chose the within-host parameter to be 1.48. This value was estimated by [2] for a tuberculosis dataset. We do not update these parameters during the inference procedure but leave them fixed. When running TransPhylo on clade A, we chose prior shape and scale parameters of the gamma distribution that gave a mean of $4-5$ years for the generation time distribution; this was to avoid enforcing short generation times by penalizing candidate transmission trees with long latent periods. However, the posterior generation times were shorter, with most cases progressing in the first two years. Case finding and management of tuberculosis is quite effective in Norway, and as such, we chose a gamma sampling distribution with mean between $2.5-3$ years; again posterior values were shorter. We chose ten different shape and scale parameters for the priors (see Table S2a). For each of these prior parameter sets, we use TransPhylo to infer an MCMC chain of length 100000. We thus obtained ten different posterior sampling and generation time distributions.

**SM**	**CM**	**DM**	SSML	BF	Best Model
GTR	Relaxed	GMRF	−16220247.863	0.000	**
HKY	Strict	GMRF	−16220255.724	7.861	
HKY	Relaxed	GMRF	−16220260.481	12.618	
GTR	Relaxed	logistic	−16220263.127	15.264	
GTR	Strict	logistic	−16220269.110	21.247	
GTR	Strict	GMRF	−16220272.238	24.376	
GTR	Relaxed	expon	−16220284.977	37.114	
GTR	Strict	expon	−16220287.084	39.222	
HKY	Strict	constant	−16220325.080	77.217	
GTR	Strict	constant	−16220325.278	77.415	
GTR	Relaxed	constant	−16220327.235	79.373	
HKY	Relaxed	constant	−16220329.503	81.641	

Table S1: *Beast model marginal likelihood results :* ***SM*** *stands for the substitution model,* ***CM****CM stands for the clock model,* ***DM*** *stands for the demographic model,* ***SSML*** *stands for the stepping stone log marginal likelihood,* ***BF*** *stands for the Bayes factor*

We obtained the posterior shape and scale parameters by fitting a gamma distribution to the posterior generation and sampling times respectively using the function $fitdistr$ in the MASS package in R. These are shown in Table S2b, along with the corresponding means. These were used as inputs for the inference of transmission events on the other clades. The results reported here are the output from the first run except where otherwise stated.

The bars in Figures S2 and S3 are the posterior generation and sampling times of the inference on clade a respectively, while the solid lines are prior distribution (blue) and the fits to the posterior distribution (red).

For a single individual, effective treatment at the time of sampling means that the time between infection and sampling is very likely to be longer than the time between infection and infecting others (the cases is treated, and uninfectious, at the time of sampling). However, for the prior densities for sampling and generation time, we take into account that many individuals do not transmit at all; they are sampled before they transmit. This will be the case in particular in countries like Norway with low TB incidence. For this reason, the mean sampling time can be shorter than the mean generation time in the priors. Of course, posteriors are not priors, and the posterior generation time can be shorter than the posterior sampling time (in fact, this is likely to be the case, because the individuals who did not infect anyone in the tree do not get assigned a posterior generation time). Furthermore, if two distributions have means m_1 and m_2, with $m_1 < m_2$, and we sample random variables x_1 and x_2 from these two distributions, it is not necessarily the case than $x_1 < x_2$. So even though the (prior) sampling time mean is less than the (prior) generation time mean, there is room for transmission. We choose variable priors for both distributions.

Posterior infection times using different priors

We compared the posterior infection times obtained using different prior parameter combinations to assess the sensitivity of inference results to parameter choices. Figures S4 show this comparison for clades a, b and e using six different prior parameter values. The priors for clade a are taken from Table S2a, while the priors for the

(a)

**(Run**	G_{sh}	G_{sc}	G mean (years)	S_{sh}	S_{sc}	S mean (years)
1	1.30	3.33	4.33	0.80	3.20	2.56
2	2.20	1.98	4.36	1.00	2.50	2.50
3	1.50	2.86	4.29	1.40	1.89	2.64
4	3.33	1.30	4.33	1.50	1.74	2.61
5	1.98	2.20	4.36	2.00	1.28	2.56
6	3.33	1.50	5.00	2.50	1.20	3.00
7	1.83	2.40	4.39	2.43	1.04	2.52
8	2.00	2.10	4.20	2.79	1.07	2.98
9	1.70	2.52	4.28	3.00	0.80	2.40
10	3.50	1.23	4.30	4.20	1.07	4.49

(b)

**Run**	G_{sh}	G_{sc}	G mean (years)	S_{sh}	S_{sc}	S mean (years)
1	1.22	0.54	0.66	0.71	0.70	0.50
2	1.89	0.49	0.93	0.79	0.76	0.60
3	1.38	0.56	0.77	1.02	0.64	0.65
4	2.35	0.54	1.27	0.94	0.83	0.78
5	2.04	0.42	0.86	1.30	0.58	0.75
6	2.77	0.47	1.3	1.45	0.55	0.80
7	1.85	0.47	0.87	1.32	0.69	0.91
8	1.89	0.51	0.96	1.54	0.56	0.86
9	1.87	0.44	0.83	1.68	0.49	0.82
10	3.11	0.43	1.34	1.67	0.62	1.03

Table S2: *Tables showing (a) prior and (b) posterior parameter values for the generation time distribution on clade a (shape is G_{sh} and scale is G_{sc}) and sampling time distribution (shape is S_{sh} and scale is S_{sc}).* The resulting means (shape times scale) are also listed. Posterior means are well below prior means, illustrating that the data are informative. The choice of longer possible time frames for both prior densities reflects an uninformative prior, to avoid overly influencing the results.

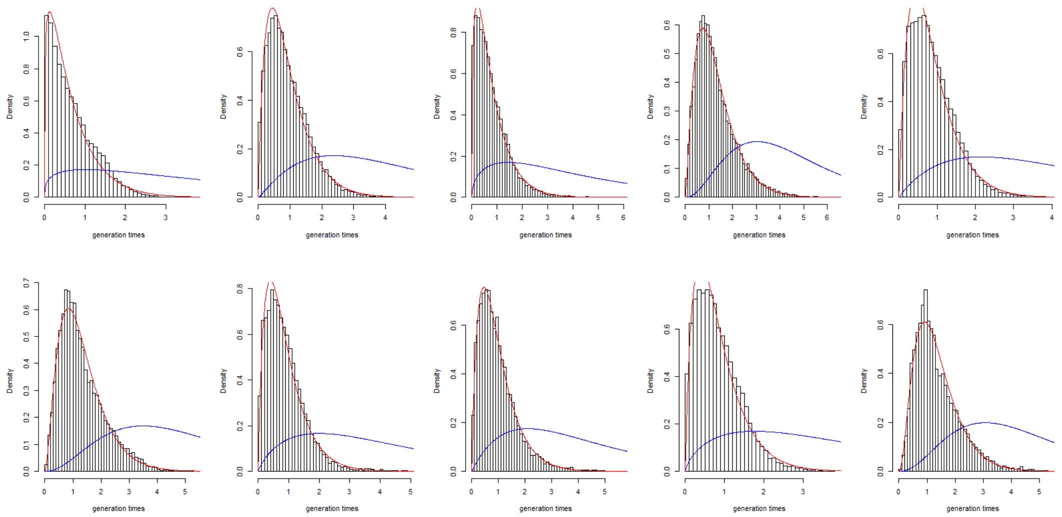

Figure S2: *Posterior generation time distribution for Clade a. The prior distribution is highlighted in blue and the posterior fits are in red.*

other clades are from Table S2*b*. Although there are slight differences in the infection time distributions inferred using different priors, the conclusion generally remains the same when these distributions are compared with arrival times in Norway.

Posterior infection times using different input timed phylogenetic trees

A key feature of TransPhylo is that it infers transmission events using a two-step procedure: obtaining a timed phylogenetic tree and inferring transmission events given this phylogenetic tree. To account for uncertainty in the phylogenetic reconstruction, the transmission inference was applied to a random sample of posterior estimated phylogenetic trees. There is also uncertainty in the choice of prior parameters for the generation and sampling time distributions. To explore sensitivity to these choices, we used a wide range of combinations that depict a tuberculosis outbreak. The inference results, although variable with different parameter choices, are consistent in the conclusions of infection pre/post arrival in Norway.

A total of 2700 timed phylogenetic trees were obtained from BEAST after thinning the BEAST MCMC chains. Figure S5 gives a visualization using multidimensional scaling of the distances between these trees, with the median (MT) and median credibility (MCC) trees highlighted. The distance used here is the metric for phylogenetic trees described recently by Kendall and Colijn [7] and implemented in the *R* package *treespace* developed by [6]. In the plot, each point represents a timed phylogenetic tree, and the distance between any pair of points approximates the distance between them given by the metric.

We extracted clade *A* from this median tree and three other random trees from the posterior set of phylogenetic trees and used the same prior parameters for the inference procedure. Figure S6 shows the posterior distributions of the infection times of the five trees plotted alongside the arrival times of the cases in the clade. Although, there are differences in the distributions as expected, with some portion of the distributions falling in regions not similar to those of the MCC tree, the conclusion the probability of infection post arrival does not change drastically across

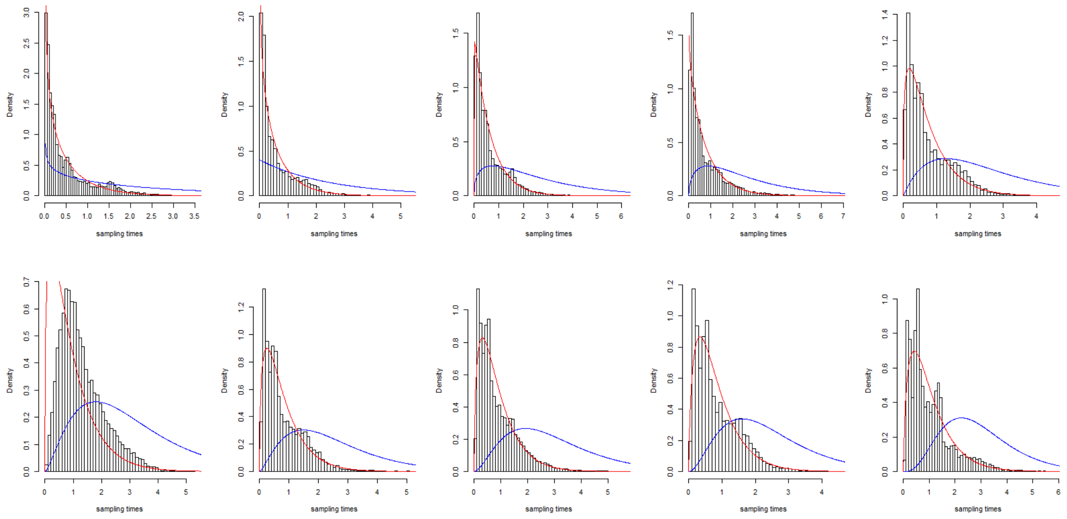

Figure S3: *Posterior sampling time distribution for Clade a. The prior distribution is highlighted in blue and the posterior fits are in red.*

the different trees.

Incorporating sputum smear results and site of infection

A penalized likelihood is just the likelihood multiplied by a penalty term or the log-likelihood with a penalty term subtracted from it. This provides a way of shifting the posterior sample of trees towards estimates that have some grounding information outside of the likelihood, analogous to prior beliefs about the transmission tree itself.Sputum smear negative patients (indicative of lower sputum bacillary load) are less infectious than smear positive patients, and thus transmit tuberculosis with less efficiency. We applied a penalty to transmission events that have smear negative infectors by multiplying the probability of the transmission tree by 0.75. As such the inferred transmission trees are pulled towards estimates that have fewer transmission events from smear-negative patients. We assume that patients with extra-pulmonary tuberculosis are only 1% as likely to transmit the disease as pulmonary tuberculosis patients, and also apply this penalization to the likelihood of a transmission tree.

We repeated the inference procedure for all the clades (start with clade A and use the posterior generation and sampling times on the other clades), using the penalized likelihood approach. The probabilities of infection after arrival in Norway for the cases are shown in Table S3. On average these probabilities are higher than those obtained using the non-penalized inference procedure.

Probability of infection prior to or after arrival

We can quantify the probability that an individual was exposed to their TB strain prior to their arrival in Norway, using the posterior times of infection. If we know the arrival time t^i_{arr} for case i, and we let the posterior time of infection density be called $L^i(\tau)$, then the probability that i was infected after arrival is just the portion of the posterior that lies above t^i_{arr}:

$$P(t^i_{inf} \text{ after } t^i_{arr}) = \int_{t^i_{arr}}^{t_{max}} L^i(\tau) d\tau. \quad (1)$$

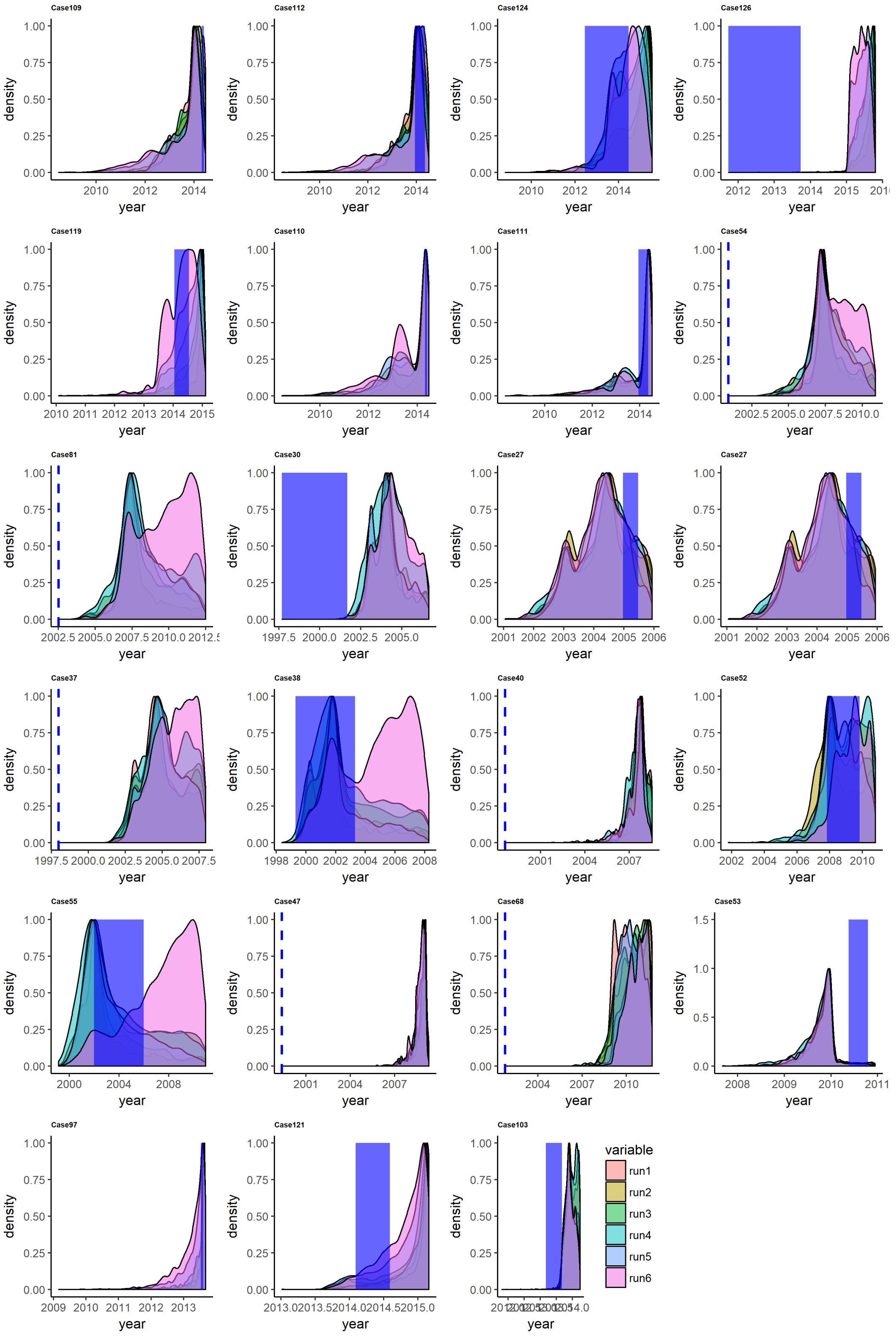

Figure S4: *Arrival times of cases in clades of interest plotted on their infection times obtained using six different prior parameter sets*

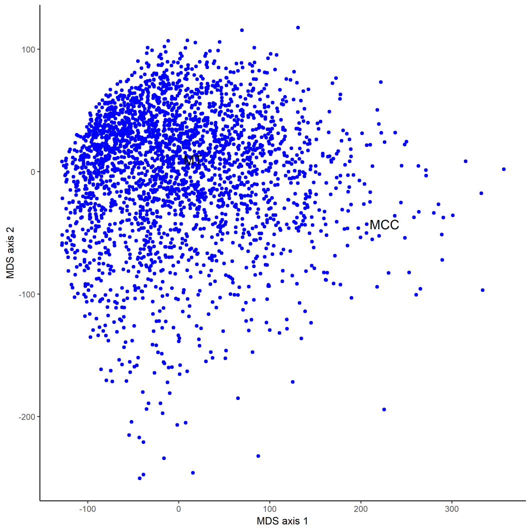

Figure S5: *Posterior distribution of timed phylogenetic trees with the maximum credibility clade tree (MCC) and median tree (MT) highlighted.*

**Case**	Clade	Country	$P_{NP}(t_{inf} > t_{arr})$	$P_P(t_{inf} > t_{arr})$	LNorway
Case 109	A	Eritrea	0.03	0.08	No
Case 110	A	Eritrea	0.11	0.30	No
Case 111	A	Eritrea	0.50	0.98	Yes
Case 112	A	Eritrea	0.22	0.40	No
Case 119	A	Eritrea	0.64	0.57	Yes
Case 124	A	Eritrea	0.88	0.81	Yes
Case 126	A	Eritrea	0.98	0.98	Yes
Case 53	B	Sudan	0.01	0.30	Yes
Case 97	B	Somalia	0.33	0.35	No
Case 121	B	Eritrea	0.78	0.80	Yes
Case 103	B	Somalia	0.98	0.98	Yes
Case 54	E	Somalia	0.99	0.97	Yes
Case 81	E	Iran	0.99	0.97	Yes
Case 30	E	Somalia	0.99	0.99	Yes
Case 27	E	Ethiopia	0.12	0.27	No
Case 37	E	Somalia	0.98	0.97	Yes
Case 38	E	Somalia	0.76	0.96	Yes
Case 40	E	Somalia	0.99	0.99	Yes
Case 52	E	Gambia	0.50	0.88	Yes
Case 55	E	Somalia	0.45	0.96	Yes
Case 47	E	Somalia	0.99	0.99	Yes
Case 68	E	Somalia	0.98	0.97	Yes

Table S3: *Table summarizing main results of study. Probabilities of infection after arrival in Norway (Not Penalized :$P_{NP}(t_{inf} > t_{arr})$ and Penalized: $P_P(t_{inf} > t_{arr})$) are shown where available. We conclude that the cases are likely infected in Norway (LNorway) based on these probabilities*

If the arrival is uncertain, and we only know that case j arrived between minimum time m_j and maximum time M_j, then we can integrate out the unknown time of

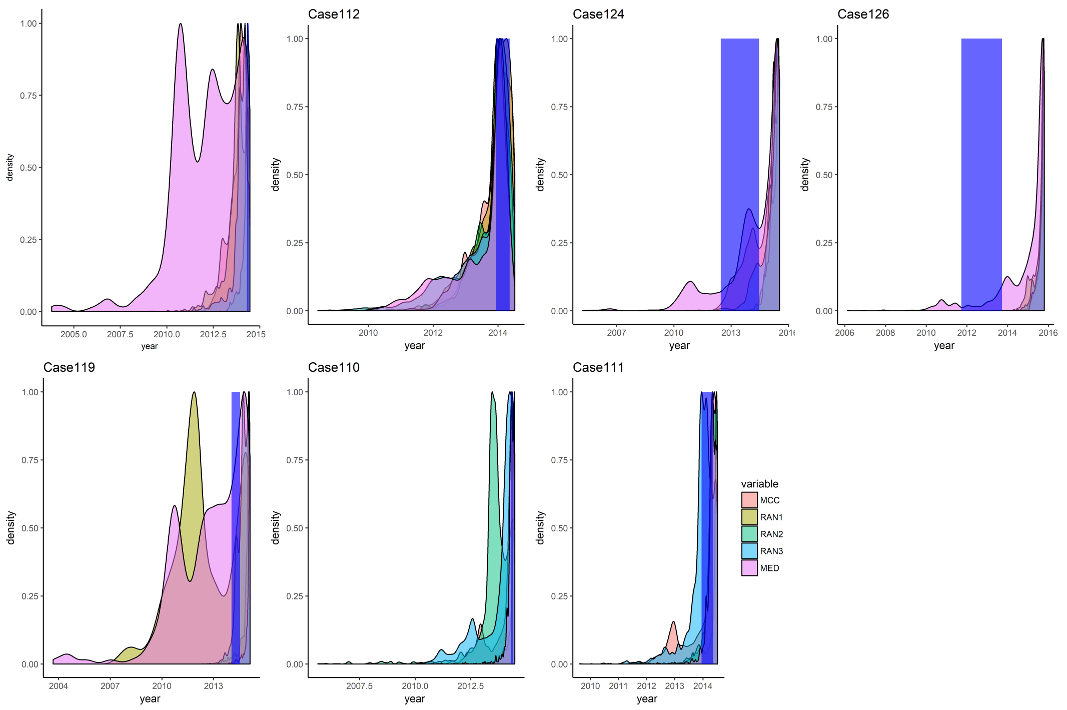

Figure S6: *Infection times of of cases in clade A using the same priors but different input trees: MCC - maximum credibility clade tree, MED - medium tree and three random trees (RAN1, RAN2, RAN3)*

arrival to find the marginal probability that j was infected after arrival in Norway:

$$P(t^j_{inf} \text{ after } t^j_{arr}) = \int_{m_j}^{M_j} P(t_{arr} = s) P(t^i_{inf} \text{ after } s) ds$$

and we use (1) to obtain $P(t^i_{inf}$ after $s)$, and a uniform distribution (blue rectangles in the Figures) for $P(t_{arr} = s)$. These probabilities are averaged over 10 inference procedures using different prior distribution parameters.

Small clusters representing putative transmission in Norway								
**Case**	**IY**	**PIN**	**P/E**	**Country**	**MPD**	**Clade**	**Transmission in Norway**	**Interpretation**
Case 14	2003	$0.5-1$	Pulm	Somalia	0	-	Probably one transmission event	Identical isolates, putative infector (Case 14) had pulmonary infection. Time of arrival consistent with transmission in Norway Different countries of origin but SNP-distance of 0 Interpretation: one transmission event
Case 107	2014	>10	Pulm	Ethiopia		-		
Case 63	2011	$1-3$	E-pulm	Somalia	8	-	No	Putative infector not infectious SNP-distance relatively high
Case 108	2011	$5-9$	Pulm	Somalia		-		
Case 61	2011	$0.5-1$	Pulm	Somalia	0	-	?	Impossible to separate infection pre/post arrival due to timing of disease and arrival
Case 62	2011	$0.5-1$	Pulm	Somalia				
Case 71	2012	>10	Pulm	Somalia	2	-	?	Impossible to separate infection pre/post arrival due to timing of disease and arrival
Case N1	1999	?	?	?		-		
Case 46	2009	$1-3$	E-Pulm	Ethiopia	14	-	Probably two transmission events	Case 98 infected in Norway based on country of origin. Based on SNP-distance and different nationality, both probably infected by unsampled infector(s) in Norway.
Case 98	2013	?	Pulm	Cote d'ivoir		-		
Case 48	2009	<1	E-Pulm	Somalia	6	-	No	Both patients had E-pulmonary TB Short time from arrival to diagnosis. Two independent imporation events
Case 79	2012	<1	E-Pulm	Somalia				
Case 77	2012	$3-5$	E-Pulm	Somalia	6	-	No	Both patients had E-pulmonary TB Two independent imporation events?
Case 89	2012	$5-9$	E-Pulm	Somalia				
Case 101	2013	$0.5-1$	E-Pulm	Somalia	4	-	?	Temporal information compatible with 101 having been infected in Norway. But not information to conclude
Case N6	2012	?	?	?		-		

Table S4: *Table summarizing additional inference:* ***PIN*** *period in Norway,* ***P/E*** *Pulmonary or Extra-pulmonary TB,* ***MPD*** *mean pairwise distance*

TransPhylo cases lacking arrival info								
**Case**	**IY**	**PIN**	**P/E**	**Country**	**MPD**	**Clade**	**Transmission in Norway**	**Interpretation**
Case 4	2012	?	Pulm	Somalia	1.2	D	Probably all infected in Norway	Based on inferred transmission chain (see Fig. S7) and country of origin: Infected in Norway by unsampled infector All patients lived in same country (See Fig. 1)
Case 5	2012	?	Pulm	Somalia		D		
Case 7	2011	?	pulm	Ethiopia		D		
Case 11	2011	?	Pulm	Somalia		D		
Case 96	2009	?	E-Pulm	Norway		D		
Case 15	2003	?	E-Pulm	Somalia	2.5	C	?	Too much info lacking, impossible to say much with certainty. However, three patients lived in same county, which could suggest transmission in Norway. See Fig. 1.
Case 16	2003	?	Pulm	Somalia		C		
Case 17	2003	?	pulm	Somalia		C		
Case 95	2013	?	Pulm	Somalia		C		
Case 28	2012	?	E-Pulm	Ghana	2.5	E	Yes, both	Country of origin makes transmission before arrival extremely unlikely
Case 29	2012	?	Pulm	Somalia		E		Infector identified in Transphylo was infected in Norway

Table S5: *Table summarizing additional inference:* ***PIN*** *means period in Norway,* ***P/E*** *means Pulmonary or Extra-pulmonary TB,* ***MPD*** *means mean pairwise distance*

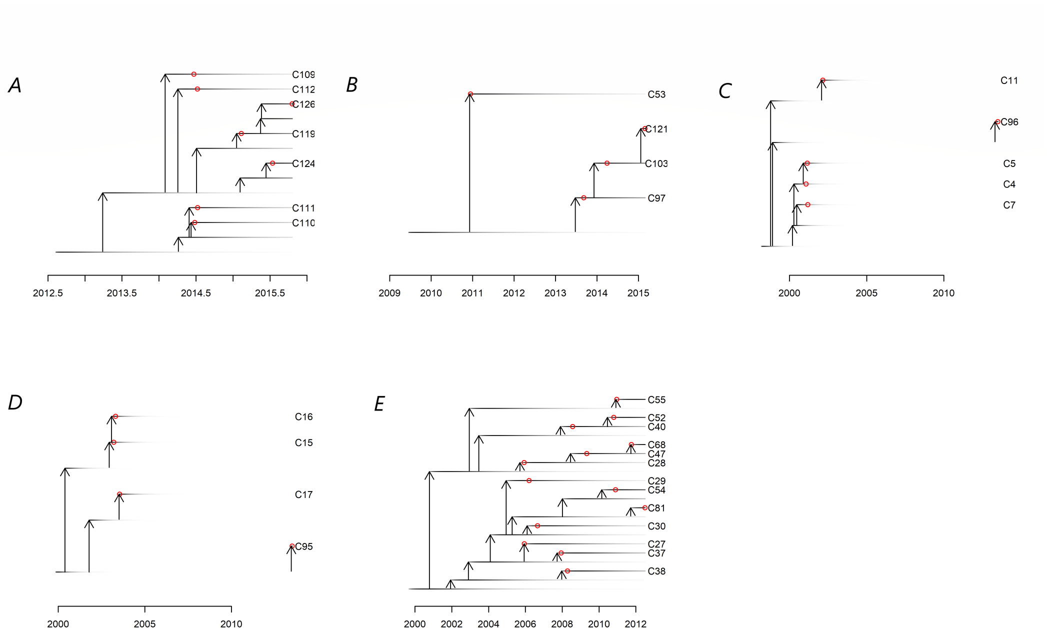

Figure S7: *Median transmission trees of the posterior distribution of transmission trees of the selected clades. Each horizontal line represents a case (sampled or inferred) with the darkness of the line representing changing infectivity over time, the red circles indicate the sampled cases and time of sampling, the vertical arrows represent transmission from case to case. The cases are illustrated as* Cn*, where* n *is the case number. Note that the infection times in these trees only reflect point estimates from the full distributions plotted in Fig. 2 of the main manuscript*

Contact tracing data

Informative data generated as part of contact tracing efforts were available for six patients belonging to clades A, B and E.

Cases 40, 47 and 81 had known TB contacts in Norway and were hence very likely infected in Norway. This is in concordance with the TransPhylo inference.

Case 119 reported to have had TB before arrival in Norway but had not been treated. With TransPhylo we inferred that the patient had been infected in Norway. For that to be correct, the patient must have been re-infection. The other option is that the patient experienced relapse of TB and that the TransPhylo inference is wrong.

Case 103 was TB-negative upon arrival based on screening. This suggests that the patient was most likely infected in Norway, which is in concordance with the TransPhylo inference.

Case 55 had been treated for TB in Norway in 2003. The isolate included in this study was from a second episode of TB in 2010. Our TransPhylo inference actually inferred that the patient was infected around 2003, even though he/she was diagnosed only in 2010. The modeling thus fits a relapse scenario well.

1 References

[1] X. Didelot, C. Fraser, J. Gardy, and C. Colijn. Genomic infectious disease epidemiology in partially sampled and ongoing outbreaks. *Molecular Biology and Evolution*, 195(4):msw075, Jan 2017.

[2] X. Didelot, J. Gardy, and C. Colijn. Bayesian inference of infectious disease transmission from whole-genome sequence data. *Molecular Biology and Evolution*, 31(7):1869–79, Jul 2014.

[3] A. J. Drummond, M. A. Suchard, D. Xie, and A. Rambaut. Bayesian Phylogenetics with BEAUti and the BEAST 1.7. *Molecular Biology and Evolution*, 29(8):1969–1973, Aug 2012.

[4] S. Duchêne, K. E. Holt, F.-X. Weill, S. Le Hello, J. Hawkey, D. J. Edwards, M. Fourment, and E. C. Holmes. Genome-scale rates of evolutionary change in bacteria. *Microbial Genomics*, 2(11):e000094, Nov 2016.

[5] C. Dye and B. G. Williams. Criteria for the control of drug-resistant tuberculosis. *Proceedings of the National Academy of Sciences*, 97(14):8180–8185, jul 2000.

[6] T. Jombart, M. Kendall, J. Almagro-Garcia, and C. Colijn. treespace : Statistical exploration of landscapes of phylogenetic trees. *Molecular Ecology Resources*, may 2017.

[7] M. Kendall, D. Ayabina, and C. Colijn. Estimating transmission from genetic and epidemiological data: a metric to compare transmission trees. sep 2016.

[8] A. Rieux and F. Balloux. Inferences from tip-calibrated phylogenies: a review and a practical guide. *Molecular Ecology*, 25(9):1911–1924, May 2016.

[9] A. Rieux and C. E. Khatchikian. tipdatingbeast: an r package to assist the implementation of phylogenetic tip-dating tests using beast. *Molecular Ecology Resources*, 17(4):608–613, Jul 2017.
